# Freehand C2 Laminar Screw Placement: Technical Note and Operative Video

**DOI:** 10.7759/cureus.5549

**Published:** 2019-09-01

**Authors:** William Clifton, Jose O Garcia, Aaron Damon, Kingsley Abode-Iyamah, Mark Pichelmann

**Affiliations:** 1 Neurosurgery, Mayo Clinic, Jacksonville, USA; 2 Neurosurgery, Mayo Clinic School of Medicine, Scottsdale, USA

**Keywords:** c2 laminar screw, freehand cervical screw, surgical video, cervical spine, vertebral artery, instrumentation safety, virtual simulation

## Abstract

The placement of C2 laminar screws is a safe and useful method of axis fixation. The freehand method of screw placement was originally described by Wright et al., and requires detailed knowledge of the C2 posterior element anatomy, relationship to vital neurovascular structures, and technical acumen. The current evidence, surgical anatomy and technical details of screw insertion are investigated and highlighted in this manuscript and surgical video.

## Introduction

Fixation of the upper cervical spine has been performed with various techniques ranging from wiring methods to polyaxial screw placement [1, 2]. Screw fixation methods for the second cervical vertebra have traditionally been performed by placement into the pars or pedicle [1]. An alternate technique of C2 fixation is the laminar screw, which is placed from the contralateral spinous process/laminar junction [3]. This technique is advantageous in that it lessens the risk of vertebral artery injury while providing a biomechanically stable means of fixation [4]. C2 laminar screw placement is generally reserved as a salvage mechanism in cases of anatomic variations in the V3 vertebral artery segment, as well as iatrogenic or traumatic disruptions of the C2 pedicle [5]. The original description of C2 laminar screw insertion utilized intraoperative line-of-sight and anatomic knowledge for intraosseous placement of C2 laminar screws [3]. This freehand technique is an effective and safe method of instrumentation without relying on image guidance systems; however it requires guided practice. At our institution, we have had success in utilizing a freehand technique for safe and efficient C2 laminar screw placement. This manuscript describes the relevant anatomy and technical details of our institution’s preferred instrumentation method using a cadaveric model.

## Technical report

The second cervical vertebra represents a specialized segment of the human cervical spine. Its primary purpose is to facilitate rotation of the head by connection to the C1 anterior arch and lateral masses [6]. The interface of the C1-2 facet joints is offset anteriorly from the subaxial cervical facets, placing the majority of the downward axial load of the head onto the C2 posterior elements. Because of this anatomic characteristic, C2 typically possesses a larger pedicle and laminar diameter than the subaxial vertebrae [7].

The bilateral C2 laminae join medially at the base of the spinous process, and extend laterally to the inferior facets. The slope and maximal diameter of the C2 lamina appear to vary significantly with gender on radiographic and cadaveric analysis [6,8]. The V2-3 vertebral artery transition occurs at the C2 foramen transversarium, which lies lateral and anterior to the C2-3 facet joint. The average antero-posterior width of the spino-laminar junction has been shown to be greater than 15 millimeters (mm) in cadaveric studies [8]. This is an important consideration in facilitating bilateral or “crossing” laminar screw placement [9].

A cadaveric specimen was acquired and the relevant anatomy and surgical technique pertaining to the freehand method of C2 laminar screw placement was highlighted in a surgical video (see Video 1). Use of three-dimensional (3D) virtual simulation software also assisted with illustration of surgical anatomy and technique.

**Video 1:** C2 freehand laminar screw operative video

Measurement of the length and diameter of the axis lamina can be performed pre-operatively with CT or MRI in order to estimate the diameter and length of screw to be inserted. The patient is placed in the prone position and head fixation is applied with the head in neutral position as in standard fashion for posterior cervical fusion. Complete exposure of the C2 posterior elements including the cranial and caudal borders is accomplished with electrocautery and curettes. If necessary, the interspinous ligament and atlantoaxial membrane are stripped from their insertion on the posterior elements of C2 in order to properly identify the extreme boundaries of the lamina.

Screw starting points are planned visually by identifying the superior and inferior borders of the spinolaminar junction in order to assess locations of screw placement in such a way that will allow the screws to be placed in a contralateral trajectory without interfering with each other’s path. Once this is accomplished, a small opening in the cortical layer of the contralateral spinolaminar junction is created with a high-speed burr. Next, a small curved pedicle probe is used to create a tract of 25-30mm angled toward the inferior facet of the contralateral C2 joint. A small ball probe is then used to check that no breach of the cortical bone of the lamina was made on either the medial portion (spinal canal) or superficial portion. The small ball probe is also used to measure the distance of the tract. A tapered tap is then used along the tract. The small ball probe is once again used to verify that there were no breaches in the created tract. A screw 3.5-4.0mm in diameter is then placed within the lamina. Once this screw is placed, the process is repeated for the contralateral screw. During placement of screws careful consideration is given to the depth at which the screws enter to prevent disruption of the C2-3 joints. Once the screws are placed, rods are cut and contoured to fit into the ipsilateral laminar screw head to join the remainder of the construct. If necessary, a lateral connector may be be used in order to properly connect the rod, although this is avoided to mitigate the potential bio-mechanical disadvantages of lateral connection [2,10].

## Discussion

The most common fixation methods of the axis involve various techniques of screw fixation, most commonly by using the C2 pars or the C2 pedicle [11]. Use of these methods provided adequate biomechanical stability, but with some risk of vertebral artery injury during screw insertion [12]. Patients with inadequate diameter, size, or deformity of the C2 pedicles pose a challenge to axis fixation. In addition, variability in the anatomy of the V3 segment of the vertebral artery coursing through the transverse foramen may put this vessel at increased risk. For situations in which the pedicle is not adequate enough for screw placement or the vertebral artery was at risk for being injured, an alternative technique was developed. Wright, et al was the first to describe the freehand placement of C2 laminar screws using a freehand method [3,13]. The authors reported success in C2 fixation using this technique a series of 10 patients with mostly traumatic pathologies. They described the potential bio-mechanical strengths of the construct as well as decreased risk of injury to the vertebral artery.

Wright’s published “crossing” technique utilizes the anatomical advantages of the C2 lamina to ensure proper placement and bio-mechanical stability [3]. Other techniques include ipsilateral insertion technique, which requires removal of the C2 spinous process and thus a decrease in potential screw length, as well as the “dorsal breach” technique which utilizes bicortical purchase and assurance that the tip of the screw has not violated the ventral lamina into the spinal canal [4,5]. There has not been a bio-mechanical study indicating advantage of one method over another.

The greatest advantage of laminar screw placement is the reduced rate of neurovascular injury in comparison to the other forms of C2 screw fixation [3,8]. An advantage of the freehand placement in particular is the ability to visualize and perform the technique without the use of intra-operative image guidance, which increases exposure of radiation to the patient and provider, and may increase operative time.

A technical challenge in placing “crossing” laminar screws is the ability to pre-plan the starting points and trajectory to ensure that the screws do not interact to the point of failure after insertion. This can be accomplished by complete and thorough exposure of the C2 posterior elements, including separation of the atlantoaxial membrane from the superior aspect of the lamina in order to gauge the extreme limits of screw placement. Cadaveric studies have indicated that the average spino-laminar diameter and cortical thickness is adequate to receive bilateral screws.

## Conclusions

The freehand method of C2 laminar screw placement appears to be a safe and effective means of atlas fixation. By demonstrating this skill in a cadaveric model and with the utilization of the latest educational technologies, the technical details of this instrumentation method can be adequately illustrated for instructional and educational purposes.
